# Ion Transfer During Ionomer Contact Electrification: Binding Affinity Controls Charging

**DOI:** 10.1002/anie.5487708

**Published:** 2026-07-21

**Authors:** John R. Hoffman, Stefan A. Freunberger, Scott Waitukaitis

**Affiliations:** ^1^ Institute of Science and Technology Austria Klosterneuburg Austria

**Keywords:** binding affinity, charge transfer, contact electrification, ion exchange, ionomer, tribocharging

## Abstract

Contact electrification occurs ubiquitously in nature, but the identity of charge carriers in most situations remains uncertain, with electrons, ions, or nanoscopic material fragments as viable candidates. One material where the species transferred seems more certain is ionomers, i.e., polymers that contain mobile ions balanced by fixed counter‐charges. When ionomers touch a neutral surface, the latter becomes charged in the sign of the mobile ion, strongly suggesting ion transfer. However, the mechanism and governing factors of transfer remain poorly understood. Here, we demonstrate that binding affinity between mobile ion and ionomer controls charge transfer with ionomers. We use ionomers with fixed anions and cations and perform ion exchange to create samples with a series of transferrable ions. We observe a strong binding‐affinity dependence for the anionic ionomer, such that mobile cations with the highest affinity transfers the least charge. Weaker, yet clear dependence was measured for the cationic ionomer, which follows the hydration free energy of the mobile anion. Using inductively coupled plasma optical emission spectroscopy (ICP‐OES), we confirm transfer of the mobile ions to the counter sample's surface. Our results confirm the identify and mechanism of charge transfer with ionomeric materials, with potential implications for contact electrification more broadly.

## Graphical abstract

The binding affinity between mobile ions and fixed ionomer groups determines the amount of charge transferred during contact electrification. Ionomers with a series of mobile alkali cations as well as mobile anions establish a clear ordering in charge transfer, where higher interaction strength leads to lower charge transfer. The transfer of mobile ions to a neutral, insulating counter surface was confirmed using chemical analysis.

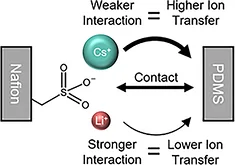

## 1 Introduction

Charge is transferred when two objects contact each other [1, 2]. This phenomenon, ‘contact electrification’ or ‘tribocharging’, has been known for millennia, but the mechanism remains poorly understood in most situations. It is established that conducting materials transfer electrons [3, 4]. However, a similar “neat and tidy” description is not established for insulating materials [5]. With insulators, even the identity of the charge carriers is unknown in most situations, with possible candidates including electrons, ions, or nanoscopic charged fragments [6]. Making matters more difficult, the process is significantly influenced by many factors [7], including surface roughness [8, 9], hydrophilicity [10, 11], and surface functionalities that impact zeta potential [12, 13, 14] or radical scavenging [15].

In one special class of semi‐insulating materials—ionomers—the situation seems more tractable. Ionomers are polymers that contain a mobile ionic species allowing them to be ionically conductive. This mobile species is balanced by a fixed charged group covalently bonded to the polymer backbone [16]. It has been experimentally demonstrated that the sign of charge transfer from ionomers matches that of the sign of the mobile ion [12, 17]. For instance, in the case of a fixed cation and a mobile anion, negative charge transferred to the counter material, and vice‐versa for a fixed anion and a mobile cation. Additionally, the transferred ion could be detected on the surface of the counter sample using surface analysis techniques, e.g., secondary‐ion mass spectrometry [18].

Ionomeric charge transfer has been evaluated as a function of mobile ion species, but the conclusion was that differences in charging resulted from a difference in polarization rather than a difference in ion transfer as a result of different ion associations [19]. This effect was described as a function of ion size due to the electrostatic field becoming stronger with decreasing ionic radius. While this may be the case, the binding affinity of fixed charges and mobile ions are also dependent on these same interactions. A Hofmeister like series has been demonstrated for polymer brushes, which identifies which mobile ions form strong association and therefore a close pair with the fixed charged groups [20, 21, 22]. We hypothesize that these interactions will play a role in the direct transfer of ions between an ionomer surface during contact, specifically, that ions with a lower binding affinity will have higher charge transfer than ions with a higher binding affinity.

**FIGURE 1:**
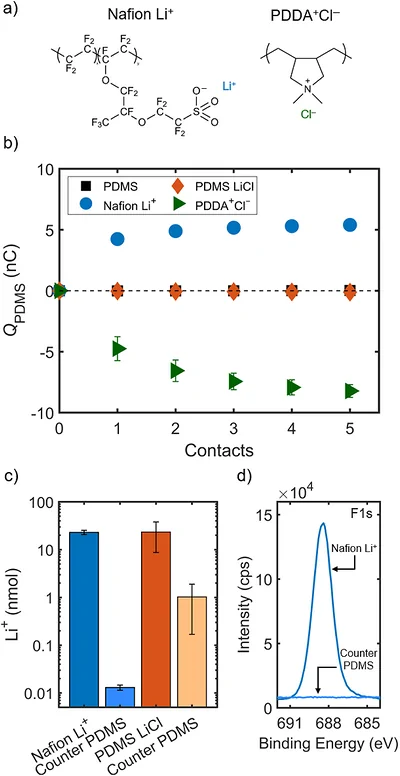
(a) Chemical structure of Nafion Li^+^ and PDDA^+^Cl^–^. (b) Charge transfer when PDMS, Nafion Li^+^, PDDA^+^Cl^–^, and PDMS with loose surface LiCl contact a bare PDMS sample. Significant charging only occurs with the Nafion Li^+^ sample and the PDDA^+^Cl^–^ sample. Moreover, the Li^+^ sample transfers positive charge to bare PDMS and the PDDA^+^Cl^–^ transfers negative charge consistent with the hypothesis that the mobile ions are transferred. The error bars represent one standard deviation of the data (*n* = 4) (c) Nanomole quantities of Li^+^ on each sample surface, as determined by ICP‐OES, demonstrates that Li^+^ transfers from Nafion Li^+^ to the counter PDMS. Li^+^ also transfers from the PDMS LiCl sample, but this does not equate to a charge transfer likely due to a balanced transfer of both Li^+^ and Cl^–^. The error bars represent one standard deviation of the data (*n* = 3) (d) F1s XPS spectrum of the counter sample, showing no detection of transferred Nafion.

Here, we probe this unaddressed aspect of ionomer contact electrification. We use Nafion, bearing bound sulfonic acid and mobile Li^+^ (Nafion Li^+^), and poly(diallyldimethylammonium chloride) (PDDA^+^Cl^–^) as commercial ionomers (Figure 1a), which we exchanged with a series of mobile cations and anions, respectively, to modulate binding affinity. Charge transfer during contact electrification was tested in a custom setup in which a linear actuator contacts the ionomer surface sample with a fixed bare polydimethylsiloxane (PDMS) counter sample inside a Faraday cup (Figure S1) [23]. The ionomers were spin coated as a 20 nm film onto plasma treated PDMS blocks. The layer thickness and their chemical identity were confirmed with ellipsometry and XPS (Figure S2) as detailed in the Methods. Both samples have a 1×1 cm flat contact surface, the contact force was fixed to 5 N and the humidity was controlled at 30% r.h. Before testing, samples were completely discharged using a custom soft x‐ray chamber.

First, four sets of samples were chosen to establish the connection between mobile charge and the sign of charge transfer (Figure 1b). The counter sample for all experiments were fresh PDMS samples with no prior contact history. First, identical PDMS samples were tested to denote the charging level that occurs due to non‐ion transfer. Next, Nafion Li^+^ and PDDA^+^Cl^–^ were separately tested against fresh PDMS samples. Finally, PDMS samples were soaked in a 10 mM LiCl solution (PDMS LiCl), dried, and contacted with fresh PDMS to evaluate the role that loose surface ions may play in ion transport.

Contacting identical PDMS samples resulted in 0.02 nC of charge transfer, which is consistent with charge transfer between identical materials [23]. A vastly different charging profile occurs when using the ionomer samples. A positive charge of +5.4 nC was measured on the counter sample PDMS after 5 contacts with Nafion Li^+^. The sign of charge on the counter sample corresponds to the mobile Li^+^ ion, consistent with Li^+^ cations transferring from the Nafion to the PDMS. Analogously, a negative charge of –8.2 nC was transferred using PDDA^+^Cl^–^, which bears a mobile anion.

The transfer of Li^+^ ions from the Nafion Li^+^ surface to the counter sample was validated and quantified using inductively coupled plasma‐optical emission spectroscopy (ICP‐OES) as detailed in the Methods. The 20 nm Nafion Li^+^ film sample contains ≈23 nmol Li^+^, of which 0.015 nmol (i.e. ≈0.6%) were found on the counter PDMS sample after five contacts (Figure 1c), directly proving Li^+^ ions do transfer during tribocharging. However, for the transferred charge of +5.4 nC, only 5.4 × 10^−5^ nmol of Li^+^ would suffice. ≈250 times as much Li^+^ was found transferred. This discrepancy may arise from discharges to the air during the contact electrification experiment. The charge level after the first contact in Figure 1b implies that the surface electric field is ≈3 MV/m, which is the nominal dielectric strength of air. During discharge some transferred Li^+^ would be reduced to metallic Li, which is not stable in ambient air and likely forms LiOH or Li_2_O with water or oxygen [24]. Therefore, only part of the Li^+^ transferred to the counter sample results in apparent charge. Another possibility could be that some Nafion transfers during contact, which would transfer Li^+^ including the balancing anion. However, x‐ray photoelectron spectroscopy (XPS) analysis of the counter sample could not detect any F while it is abundant in Nafion (Figure 1d). Similarly, we found no trace N as a signature for PDDA^+^ (Figure S3). Both measurements confirm that material transfer play little role in the overall charge transfer.

To examine the possibility of loosely bound surface salt transferring during contact, PDMS samples that had LiCl deposited on them were contacted and resulted in –0.12 nC after 5 contacts. ICP‐OES measurements have shown that 23.3 nmol of Li^+^ were on the initial sample and 1.03 nmol Li^+^ were transferred (Figure 1c). While the available Li^+^ is similar to the Nafion Li^+^ sample, ≈80‐fold increase in Li^+^ was transferred in accord with both ions being weakly bound. However, this transfer did not correspond to any net charge transfer.

**FIGURE 2:**
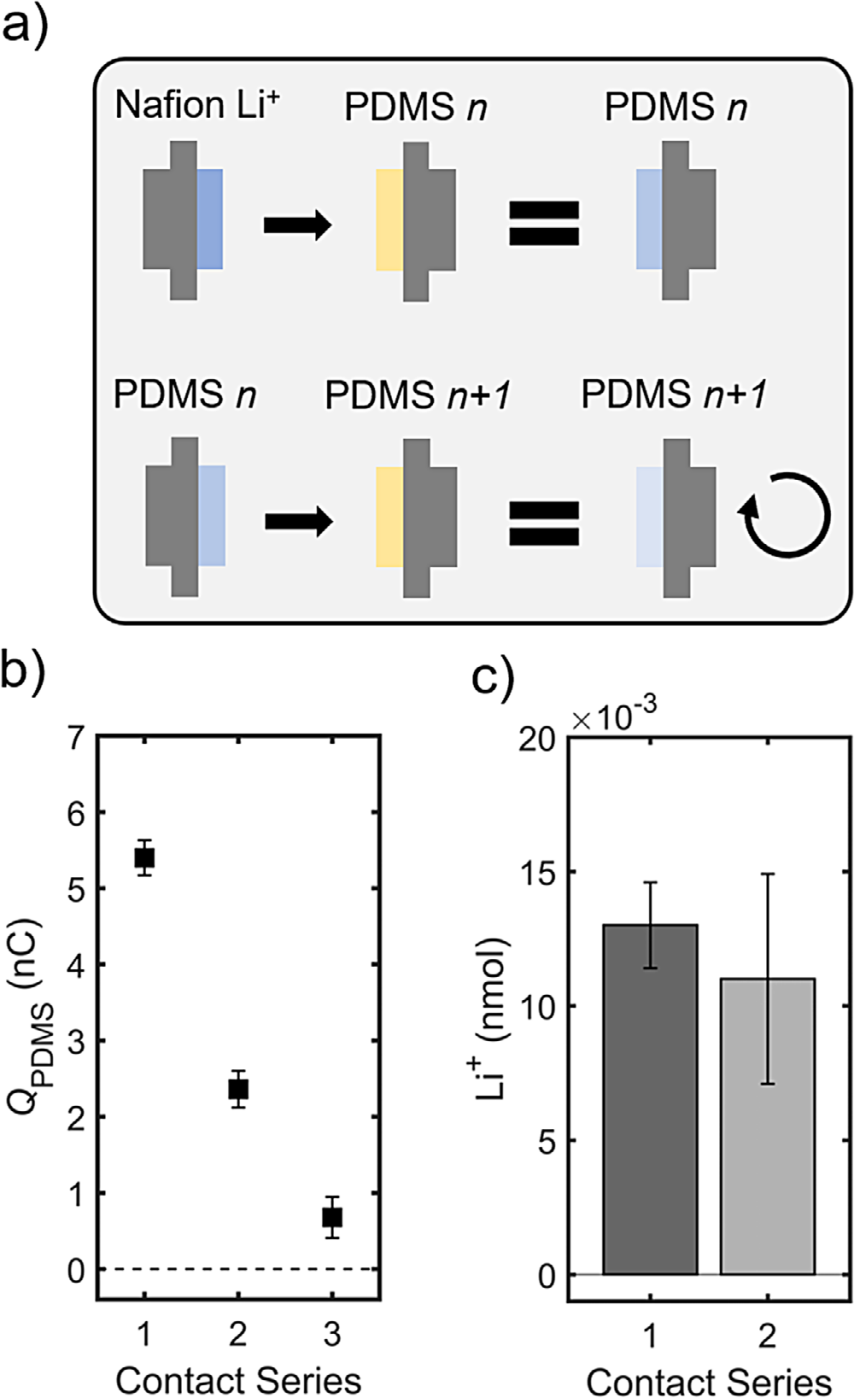
Probing ion transfer from sequential PDMS contact. (a) Nafion Li^+^ initially makes contact with the first PDMS sample, labeled *n*. A total of 5 contacts are performed. PDMS *n* is then contacted against a fresh PDMS sample, labeled *n* + 1. A total of 20 contacts are performed between PDMS *n* and PDMS *n* + 1. This process repeats to create a series of PDMS samples. (b) Charge transfer of PDMS 1, PDMS 2, and PDMS 3 after following the contact series steps shown in panel a. The error bars represent one standard deviation of the data (*n* = 4) (c) Li^+^ content on PDMS after contact series 1 and 2 were measured via ICP‐OES demonstrating that ion transfer occurs beyond contact with just the Nafion surface. The error bars represent one standard deviation of the data (*n* = 3).

We additionally examined how ions transferred to a first bare PDMS would further transfer when contacted to a second bare PDMS as shown schematically in Figure 2a. First, the ionomers were contacted with a first PDMS sample, denoted as PDMS 1, resulting in the as discussed ion transfer. This charge after 5 contacts is reported as Contact Series 1 in Figure 2b. After this contact, another PDMS sample, denoted PDMS 2, was placed in the testing device and contacted against PDMS 1. PDMS 1 was not discharged after contact with the ionomer sample. 20 contacts were made between PDMS 1 and PDMS 2, the charge after the twentieth contact is reported as Contact Series 2. This was repeated for one additional series of PDMS samples.

After contact with Nafion Li^+^, the total charge on PDMS 1 was 5.4 nC. PDMS 1 was then contacted with PDMS 2, which resulted in a transfer of 2.4 nC to PDMS 2 after 20 contacts were made. The number of contacts was increased from 5 to 20 as saturation did not appear to occur after 5 contacts. When PDMS 2 was contacted with PDMS 3, the resulting charge was 0.68 nC. This further decrease is similar in value to pure PDMS versus PDMS contacts which follow the same protocol of 0.28 nC (Figure S4). A similar process was performed for the PDDA^+^Cl^–^ samples (Figure S4), which initially charged the PDMS 1 to –8.2 nC. Contact with PDMS 2 resulted in –2.43 nC of charge measured on PDMS 2. Similarly to the Nafion Li^+^ case, an additional contact with PDMS 3 results in a surface charge of 0.22 nC, which is very similar to the PDMS case.

During contact between PDMS 1 and PDMS 2, a key difference exists between this contact and the original contact between the ionomer and PDMS. In the ionomer case, the charge transfer has been linked to electron donation due to the potential formed between the fixed and mobile charges on the ionomer surface, resulting in electron transfer between surfaces [19]. After contact with PDMS 1, mobile ions are on the PDMS surface, but the lack of fixed charge groups means that no ionic polarization can develop between the surface Li^+^ ions and the neutral PDMS surface. As the source of Li^+^ on the PDMS 1 surface is much lower than that of the ionomer, a lower transfer threshold to PDMS 2 is achieved. The presence of Li^+^ on the surface of PDMS 2 was also measured using ICP‐OES (see Methods). A value of 0.010 nmol of Li^+^ was measured after 20 contacts as shown in Figure 2c. When compared to the charge transfer of 2.4 nC, the measured Li^+^ is ≈400‐fold the value of Li^+^ needed to account for the charge. The decrease in Li^+^ content from PDMS 1 to PDMS 2 (15% decrease) does not fully match the decrease in charge transfer that occurs (56% decrease). This difference may arise due to the Li^+^ detection limit of the ICP, which requires a large quantity of samples to be concentrated in order for Li^+^ detection. This results in the larger error bars seen in Figure 2c. When an additional contact series is made with PDMS 3, the charge transfer is further reduced and is more similar to fresh PDMS versus PDMS contact. We were unable to measure the Li^+^ content on PDMS 3 due to sample detection limits.

The results so far confirm that the mobile charge of the ionomer determines the sign of the transferred charge. We further analyze this process by associating different mobile ions with the fixed ionomer groups to determine the role that binding affinity has on ion transfer. After exchanging the Li^+^ from the Nafion with H^+^ by soaking in deionized water, Na^+^, K^+^, Rb^+^, and Cs^+^ were introduced by soaking the samples in 10 mM of their Cl^–^ solutions. That the Nafion film remained on the surface was checked by XPS (Figure S5). Removal of Li^+^ and uptake of the new cations was confirmed by ICP‐OES (Figures S6, S7); for Na^+^ and K^+^ also the amount incorporated into the Nafion film was measured. Li^+^, Na^+^, and K^+^ amounts were 23.0, 17.4, and 11.8 nmol per sample, respectively (Figure S7), while Rb^+^ and Cs^+^ could not be measured with the available ICP‐OES. Given that only ≈1/1000 of the available Li^+^ was transferred (Figure 1c), these differences in cation concentrations are expected not to influence charge transfer profoundly.

**FIGURE 3:**
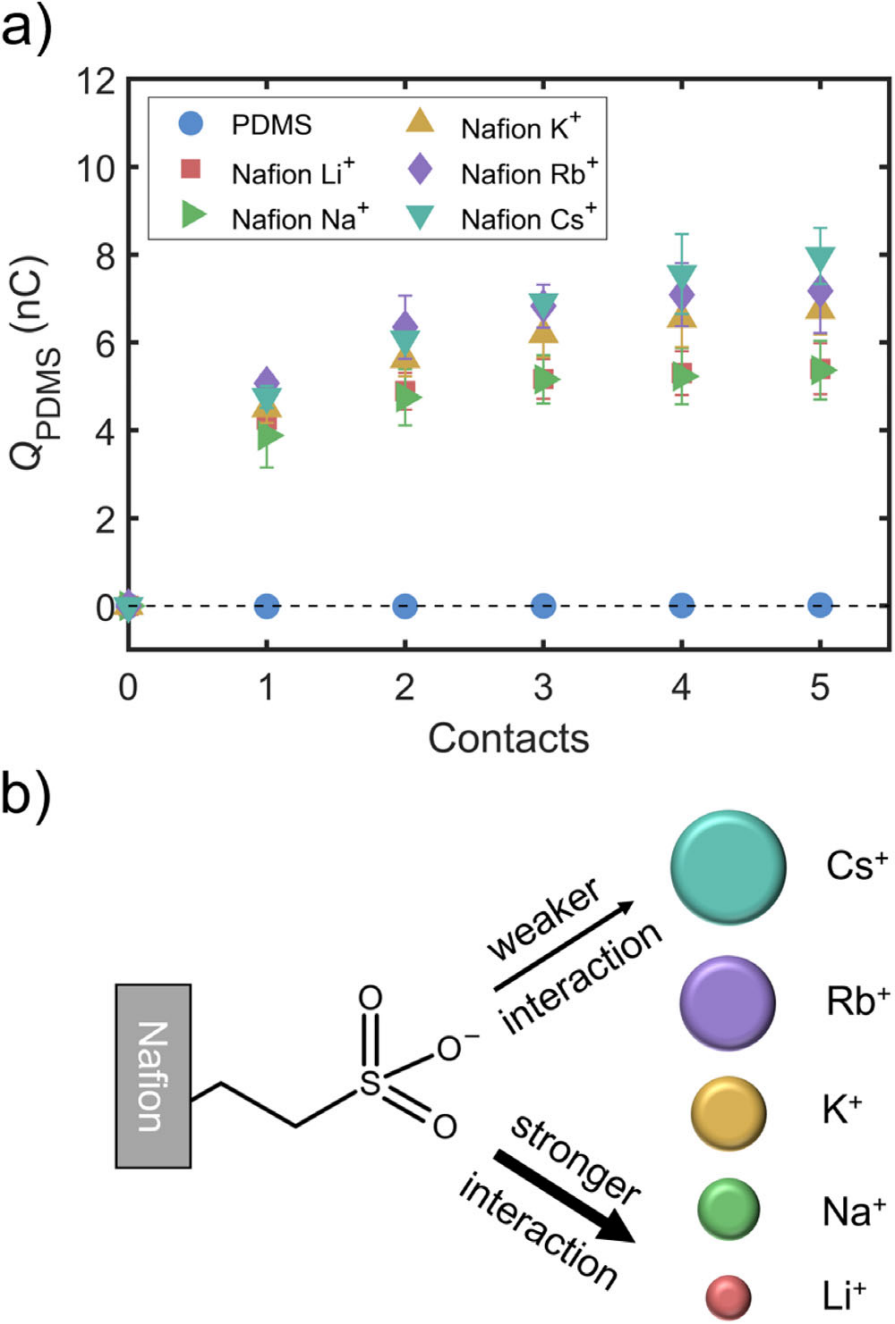
(a) Nafion ion exchanged charge transfer results for mobile ions of Li^+^, Na^+^, K^+^, Rb^+^, and Cs^+^. The error bars represent one standard deviation of the data (*n* = 4) (b) Ordering of relative interaction strength between mobile ions and sulfonic acid groups based on hard‐soft acid‐base principles.

Results for contact charging after 5 contacts with these ion‐exchanged samples are shown in Figure 3. A clear trend is observed with charge dropping from Cs^+^ towards Li^+^. Transferred charges were 8.0 nC for Cs^+^, 7.2 nC for Rb^+^, 6.7 nC for K^+^, and 5.4 nC for both Na^+^ and Li^+^. The transfer of Na^+^ and K^+^ to the counter surface was also measured with ICP‐OES (Figure S8) and each found to be ≈2.0 nmol, which is higher than the 0.01 nmol in the Li^+^ case, but similar to the PDMS LiCl case. The scale of the charge transfer for these samples in Figure 3a shows that, like Li^+^, the surface electric fields are comparable to the dielectric strength of air. In addition to this, we speculate that surface ions may not have been fully removed during rinsing after the ion exchange.

It is expected that ordering of the charge transfer correlates to interactions between the sulfonic acid groups and the mobile ion. Sulfonic acid groups are characterized as hard acids [25], which favor interactions with hard cations (Li^+^ and Na^+^) compared to soft cations (Cs^+^ and Rb^+^). The interaction trend of Li^+^ > Na^+^ > K^+^ > Rb^+^ > Cs^+^ has been evaluated by monitoring the shift of the –SO_3_^−^ symmetric stretch vibration as a function of mobile ion species [26]. Our results follow this trend with the highest charge transfer occurring with Cs^+^ and Rb^+^, which has the weakest interactions. The lowest charge transfer occurred with Li^+^ and Na^+^, which has the strongest interactions. These results clearly indicate that binding affinity plays an important role in the transfer of charge in ionomer contact electrification.

**FIGURE 4:**
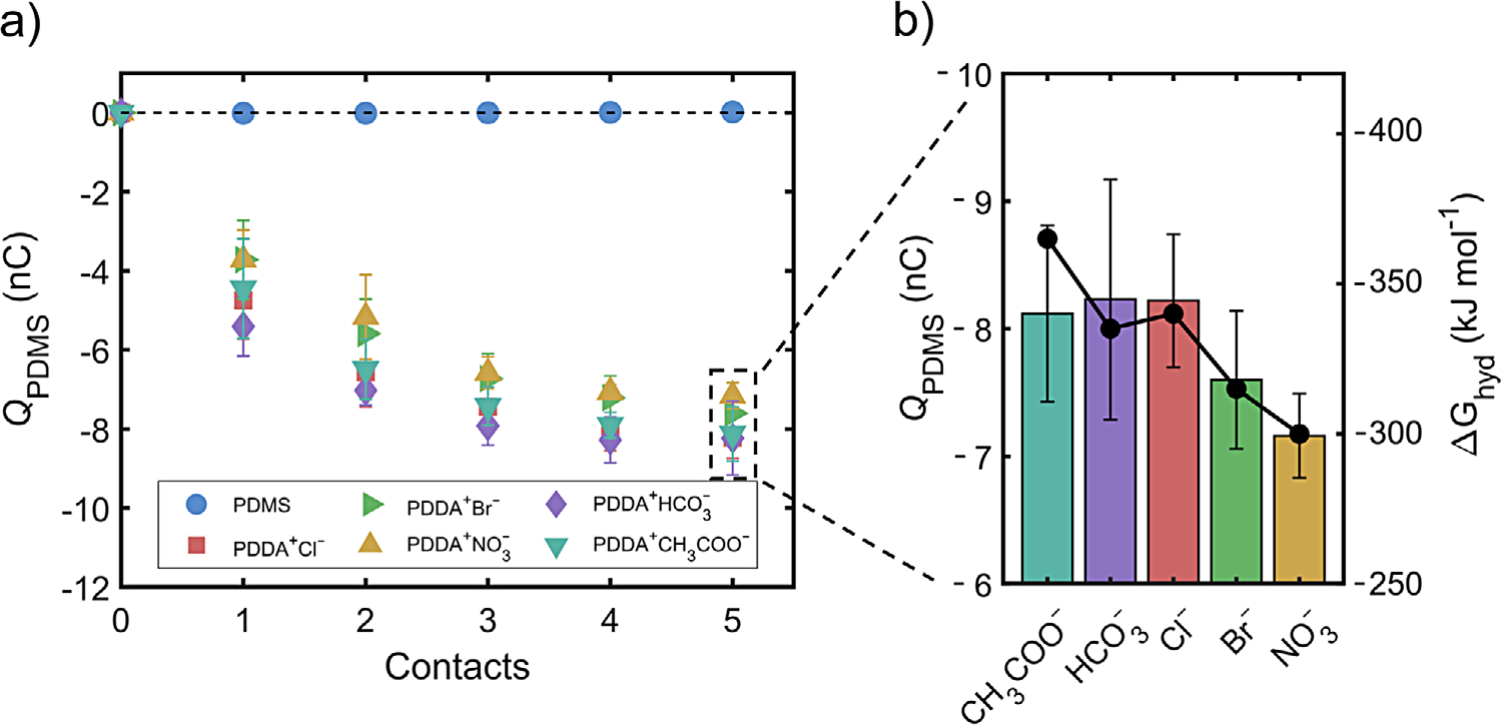
(a) PDDA^+^ ion exchanged charge transfer results for mobile ions of Cl^–^, Br^–^, NO_3_^–^, HCO_3_^–^, and CH_3_COO^–^. The error bars represent one standard deviation of the data (*n* = 4) (b) The free energy of hydration ($\Delta G_{\text{hyd}}$) of each ion, where a more negative free energy of hydration matches a higher charge transfer seen during contact electrification.

Similarly, PDDA^+^ samples with a range of anions were prepared. Cl^–^ was first exchanged with OH^–^ in DI water, followed by soaking in 10 mM solutions of NaBr, NaNO_3_, NaHCO_3_, and CH_3_COONa. Initial removal of Cl^–^ from the PDDA sample was confirmed by XPS (Figure S9). After drying, contact electrification measurements were performed as shown in Figure 4.

The trend for anion transfer from PDDA^+^ depends much less on the mobile ion compared to the Nafion case. Charge transfer follows in decreasing order Cl^–^ and HCO_3_^–^ (–8.2 nC each), CH_3_COO^–^ (–8.1 nC), Br^–^ (–7.6 nC), and NO_3_^–^ (–7.2 nC). While the differences between the ions are low, there is still an ordering. There are many possible factors that may control this ordering. Ionic radii, as used in the Nafion case, is one possibility, but the PDDA^+^ results contradict this with higher charge transfer occurring for the smaller Cl^–^ anion than the larger Br^–^. The Nafion case has a delocalized charge across its fixed group, which differs from the highly localized charge of the quaternary amine. This may account for why different factors control charging in this case. One parameter that matches the order is the hydration free energies of the anions, which are plotted along with the charge transfer result in Figure 4b. The values for the hydration free energies (Δ*G*_hyd_) have been taken from literature and define how strongly the ion interacts with water [27]. This interaction is controlled by the hydration free energy of each ion, where stronger hydration means stronger interactions with water than with the quaternary amine [27]. Hydration stabilizes CH_3_COO^–^, Cl^–^, and HCO_3_^–^ most strongly, which can disrupt the ionic attraction between the mobile ions and the quaternary amine group. As our experiment does not occur in the hydrated state, humidity in the environment (controlled at 30% RH), may begin to solvate and disrupt the ionic interactions. This weakened anion‐to‐amine interaction corresponds to the ions having the highest charge transfer. The lower hydration free energies of Br^–^ and NO_3_^–^ correspond to stronger anion‐to‐amine interaction and decreasing charge transfer. These results further confirm that binding affinity controls ion transfer.

In summary, we could quantitatively link the ion‐to‐ionomer interaction strength to the magnitude of ionic charge transferred during contact electrification between ionomer and an insulating surface (PDMS). Ion transfer to the counter sample surface was confirmed using ICP‐OES. Transferred ions can further transfer net charge to fresh insulator surfaces, which indicates that the ions remain in the ionized state. A clear dependence on binding affinity was seen with Nafion as a function of the mobile alkali metal ion. Larger, softer mobile ions (such as Rb^+^ and Cs^+^) interact weakly with the hard sulfonate in Nafion, which results in higher charge transfer compared to smaller, harder mobile ions (such as Li^+^ and Na^+^). Weaker, yet clear dependence was measured with the PDDA^+^ ionomer, likely due to more localized charge on the quaternary ammonium and smaller differences in interaction. The measured charge transfer followed the order of the mobile ion's hydration free energy, which suggests that ions with a more negative hydration free energy more easily become hydrated from humidity in the environment and interact more weakly with the fixed ion, resulting in more charge transfer. These results help present a clearer picture of ionomer‐based contact electrification.

## Author Contributions

**John R. Hoffman**: methodology, data curation, writing – original draft, investigation, conceptualization, formal analysis. **Stefan A. Freunberger**: writing – review and editing, conceptualization, supervision, methodology, formal analysis. **Scott Waitukaitis**: conceptualization, supervision, writing – review and editing, methodology, formal analysis.

## Conflicts of Interest

The authors declare no conflicts of interest.

## Supporting information



Methods and additional data are available in the supplementary material of this article.**Supporting File**: anie73605‐sup‐0001‐SuppMat.docx.

## Data Availability

The data that support the findings of this study are available from the corresponding author upon reasonable request.
