# Supplementary material for: Ion Transfer During Ionomer Contact Electrification: Binding Affinity Controls Charging
Source: Angew Chem Int Ed Engl. 2026 Jul 21;65(36):e5487708. doi: 10.1002/anie.5487708 (PMC13528513; doi:10.1002/anie.5487708)
Supplement: Supplementary file 1 [file ANIE-65-e5487708-s001.docx]

Supporting Information for

Ion Transfer During Ionomer Contact Electrification: Binding Affinity Controls Charging

John R. Hoffman, Stefan A. Freunberger, Scott Waitukaitis

Corresponding author: Scott Waitukaitis, scott.waitukaitis@ist.ac.at

Institute of Science and Technology Austria

Am Campus 1, 3400 Klosterneuburg, Austria

**The file includes:**

Materials and Methods

Figs. S1 to S9

Materials and Methods

All chemicals were purchased form Sigma-Aldrich unless noted otherwise. Sylgard 184 (Dow) was used for the preparation of polydimethylsiloxane (PDMS) samples. Lithiated Nafion (Ion Power) and poly(diallyldimethylammonium chloride) (PDDA^+^Cl^–^) (Sigma-Aldrich) ionomer solutions were used to introduce bound charge groups to the system. Salts utilized for cation exchange (LiCl, NaCl, KCl, RbCl, and CsCl) and for anion exchange (NaCl, NaBr, NaNO_3_, NaHCO_3_, and CH_3_COONa) were acquired from Sigma-Aldrich and solutions were prepared using deionized water (Milli-Q EQ 7000).

Sample Preparation

PDMS samples were prepared using the Sylgard 184 standard preparation protocol. A 10:1 mixture of elastomer and crosslinker were mixed and poured into a metallic weighing dish to a thickness of 3 mm. PDMS samples were degassed for 1 h and cured at 80 °C for 24 h. 1 cm × 1 cm samples were cut from the PDMS slab using a custom-fabricated stencil and a razor blade.

Ionomer functionality was introduced via spin coating. First, PDMS samples were plasma cleaned at low power (~80W) for 30 s. A 0.65 wt% solution of Nafion Li^+^ in ethanol, and a 1 wt% solution of PDDA^+^Cl^–^ in ethanol were spin coated onto the cleaned PDMS at 3000 RPM for 3 min., respectively, for preparation of the desired ionomer sample. The thickness of each ionomer coating was verified using ellipsometry (Sentech SENPRO) by performing the same spin coating process on a clean Si wafer. Ionomer introduction was also confirmed using X-ray photoelectron spectroscopy (XPS) (Thermo Scientific ESCALAB QXi) of the PDMS spin coated samples. Specific functional groups for both Nafion and PDADMAC were measured and confirmed the ionomer introduction.

Ion Exchange and Contact Electrification Experiments

Spin coated Nafion Li^+^ samples were ion exchanged by first soaking in deionized (DI) water for 2 h to remove initial Li^+^ ions. The DI water was exchanged after 1 h to a fresh DI solution. This process fully removed all Li^+^ present in the Nafion film and was confirmed using inductively coupled plasma-optical emission spectroscopy (ICP-OES) (Shimadzu ICPE-9800). This is shown in Figure S6. This DI water soak did not affect the presence of the Nafion polymer, as XPS scans of the surface before and after soaking in DI water were unchanged, shown in Figure S5. This was followed by soaking in a 10 mM metal chloride solution of the desired cation for 1 h. Samples were gently rinsed in DI water after soaking to remove any loose ions from the surface. The concentration of Li^+^, K^+^, and Na^+^ were confirmed also with ICP-OES, as shown in Figure S7. 1 wt% nitric acid with trace metals was used as the ICP-OES testing solution. Ion exchanged Nafion samples were soaked in the 1 wt% nitric acid solution for 24 h to release all mobile ions into the testing solution. The concentration of Rb^+^ and Cs^+^ ions were not measured due to limitations in detecting these elements in ICP-OES.

To validate and quantify the transfer of ions from Nafion Li^+^ to the counter sample, the fresh Nafion Li^+^ sample and the PDMS counter sample after five contacts were soaked in 1 wt % nitric acid. This highly acidic solution disassociates all the Li^+^ ions from the sulfonic acid groups. These nitric acid solutions can be measured directly by ICP-OES. Eight sets of samples (one Nafion Li+ pressed against one PDMS) were combined and used for one ICP-OES sample because the concentration of Li^+^ measured on the counter sample was below the detection limit when only one sample was used. Twenty sets of samples, where used to measure the Li^+^ transfer from PDMS 1 to PDMS 2 as the concentration of Li^+^ in the ICP-OES sample was too dilute with just eight samples.

This was repeated for cations of Na^+^ and K^+^. Eight sets of samples of Nafion Na^+^ and Nafion K^+^ pressed against PDMS were combined to measure the amount of transferred cations for the ion exchanged samples. These results are shown in Figure S8.

Spin coated PDDA^+^Cl^–^ samples were also ion exchanged in a similar manner. Samples were soaked in DI water for 2 hours, with the water solution exchanged after 1 h. This process fully removed Cl^–^ from the sample surface and was confirmed using XPS, as shown in Figure S9. Soaking in 10 mM salt solutions introduced the desired anions.

A series of 4 samples for each mobile cation and anion were prepared and tested against fresh PDMS to determine the contact electrification effects. Samples were affixed to 3D-printed holders using a PDMS mixture of thiol end group modified PDMS, vinyl terminated PDMS, and a 2,2-dimethoxy-2-phenylacetophenone photoinitator. This glue was painted onto the holder surface, the PDMS samples were placed on top, and UV light was exposed to the sample for 2 minutes to crosslink the glue with the bottom side PDMS.

Charge measurements were carried out using a Keithley 6514 electrometer connected to a Faraday cup. A linear actuator moved the ionomer samples towards the counter sample located stationary within the Faraday cup. The counter samples were pristine, non-modified PDMS samples. Fresh samples were used for each measurement and were not reused due to the possibility that the transfer of ions during contact could impact further measurements. The charge on this pristine PDMS was measured over time during a series of five contacts. A diagram of the experimental setup is shown in Figure S1.

Quantifying Li^+^ Amount from Surface Charge

The surface Li^+^ can be calculated from the contact electrification result using algebra. First, the measured surface charge of nC cm^–2^ is converted to elementary charges using the following conversion factor: 1 nC = 6.24 × 10^9^ e. This is then converted to mol cm^–2^ by using Avogardo’s constant: $N_{A}$ : 6.022 × 10^23^ e mol^–1^.

**Figure S1.** Scheme of the experimental setup used to measure charge transfer.

**Figure S2.** a) XPS spectra of PDMS, b) Nafion Li^+^, and c) PDDA^+^Cl^–^. Specific peaks form corresponding to the spin coated material, including a F1s peak which is only present in the fluorinated backbone of Nafion and the N1s peak only present in the amine group of PDDA^+^Cl^–^.

**Figure S3.** N 1s XPS spectra of PDDA^+^Cl^–^ surface and counter surface to demonstrate the material transfer does not occur.

**Figure S4.** Results for PDMS vs PDMS and PDDA^+^ Cl^–^ ion series. The error bars represent one standard deviation of the data (n = 4).

**Figure S5.**: a) XPS spectra of Nafion Li^+^, and b) PDDA^+^Cl^–^ post DI soak. The characteristic peaks for the spin coated materials remain.

**Figure S6.** a) Calibration curve of Li^+^ standards at 670.784 nm. b) Li^+^ concentration present in fresh PDMS, spin coated Nafion on PDMS, fresh PDMS soaked in DI water for 2 hours, and Nafion on PDMS soaked in DI water for 2 h. The DI water was exchanged after 1 h and replaced with a fresh solution. Complete disassociation of Li^+^ from the Nafion is achieved within the 2 h soak, indicating that it is ready for further exchange with different mobile ion species. The error bars represent one standard deviation of the data (n = 3).

**Figure S7.** Ion amount of Nafion Li^+^, Nafion Na^+^, and Nafion K^+^ after ion exchange. The error bars represent one standard deviation of the data (n = 3).

**Figure S8.** Ion amount of Li^+^, Na^+^, and K^+^ on PDMS 1 after contact with Nafion ionomer. The error bars represent one standard deviation of the data (n = 3).

**Figure S9.** Cl 2p XPS spectra of PDDA^+^Cl^–^ before and after ion exchange show no detection of Cl^–^, indicating that full disassociation occurs.
